# Associations between depression subtypes, depression severity and diet quality: cross-sectional findings from the BiDirect Study

**DOI:** 10.1186/s12888-015-0426-9

**Published:** 2015-03-04

**Authors:** Corinna Rahe, Bernhard T Baune, Michael Unrath, Volker Arolt, Jürgen Wellmann, Heike Wersching, Klaus Berger

**Affiliations:** Institute of Epidemiology and Social Medicine, University of Münster, Münster, Germany; Discipline of Psychiatry, School of Medicine, University of Adelaide, Adelaide, SA Australia; Department of New Public Health, School of Human Sciences, Osnabrück University, Osnabrück, Germany; Department of Psychiatry and Psychotherapy, University of Münster, Münster, Germany

**Keywords:** Depression, Depression subtypes, Depression severity, Nutrition, Diet, Diet quality, Lifestyle

## Abstract

**Background:**

Depression is supposed to be associated with an unhealthy lifestyle including poor diet. The objective of this study was to investigate differences in diet quality between patients with a clinical diagnosis of depression and population-based controls. Additionally, we aimed to examine effects of specific depression characteristics on diet by analyzing if diet quality varies between patients with distinct depression subtypes, and if depression severity is associated with diet quality.

**Methods:**

The study included 1660 participants from the BiDirect Study (n = 840 patients with depression, n = 820 population-based controls). The psychiatric assessment was based on clinical interviews and a combination of depression scales in order to provide the classification of depression subtypes and severity. Diet quality scores, reflecting the adherence to a healthy dietary pattern, were calculated on the basis of an 18-item food frequency questionnaire. Using analysis of covariance, we calculated adjusted means of diet quality scores and tested differences between groups (adjusted for socio-demographic, lifestyle-, and health-related factors).

**Results:**

We found no differences in diet quality between controls and patients with depression if depression was considered as one entity. However, we did find differences between patients with distinct subtypes of depression. Patients with melancholic depression reported the highest diet quality scores, whereas patients with atypical depression reported the lowest scores. Depression severity was not associated with diet quality.

**Conclusions:**

Previous literature has commonly treated depression as a homogeneous entity. However, subtypes of depression may be associated with diet quality in different ways. Further studies are needed to enlighten the diet-depression relationship and the role of distinct depression subtypes.

**Electronic supplementary material:**

The online version of this article (doi:10.1186/s12888-015-0426-9) contains supplementary material, which is available to authorized users.

## Background

With an estimated prevalence of about 300 million cases of major depressive disorder (MDD) worldwide, depression has become a major public health concern and accounts for a considerable part of the global burden of disease [1]. In recent years, the relationship between depression and modifiable lifestyle factors, such as diet, physical activity, smoking, or sleep, has been a key area of interest [2-4]. In this context, growing evidence suggested a complex relationship between diet and depression: Diet may have impact on the development and the course of depression, and cases with depression in turn may develop unhealthy dietary habits [4-6]. Overall, findings indicate that nutrition is relevant not only for physical but also for mental health [7].

Prior studies on the diet-depression relationship mostly examined the effects of single nutrients or foods, whereas current research focuses on the effects of dietary patterns and overall diet quality [8,9]. Recent systematic reviews and meta-analyses indicated that unhealthy Western diets (e.g., rich in fast food, meat, refined grains, and sweets) may increase the risk of depression, whereas healthy high-quality diets (e.g., rich in fruits, vegetables, and fish) may have a protective effect [10-13]. Studies examining the effects of depression on lifestyle habits also suggested that depressed subjects show more unfavorable health behaviors than non-depressed subjects [14]. Various studies reported that the presence of depressive symptoms is associated with unhealthy food choices, for instance, higher intakes of fast food [15] and high-calorie sweets [16] as well as a lower consumption of fruits and vegetables [17]. However, research on the effect of depression on the overall diet quality is still scarce and yielded conflicting results up to this point [10]. Since unhealthy lifestyle habits such as poor diet may promote the development and the progression of common somatic comorbidities of depression, e.g., cardiovascular diseases (CVD), the investigation of lifestyle factors in patients with depression should receive greater attention [6,18].

The majority of previous literature examined the diet-depression relationship by assessing depressive symptomatology using self-report depression scales, as these are time- and cost-efficient [19]. However, the gold standard method in depression assessment is the clinical interview [19,20]. Up to now, only few studies examined dietary habits in samples with a clinical diagnosis of depression. Another unresolved aspect in this research area is the role of specific characteristics of depression, such as depression severity or diagnostic subtypes, on diet. Previous studies commonly treated depression as a homogeneous entity. This approach has been increasingly criticized, as depression is a phenotypically and biologically heterogeneous illness [21-23]. In particular, the melancholic and the atypical subtype strongly differ in their symptomatology including dietary aspects. Specifically, melancholic depression is related to reduced appetite and weight loss, whereas atypical depression is related to increased appetite and weight gain [23]. Hence, it could be expected that dietary patterns and diet quality vary across distinct subtypes of depression.

The aim of this study was to investigate differences in overall diet quality between patients with a clinical diagnosis of depression and healthy population-based controls. Additionally, we aimed to examine effects of specific depression characteristics on diet by examining if diet quality varies between patients with distinct subtypes of depression, and if depression severity is associated with diet quality.

## Methods

### Study population

The BiDirect Study is an ongoing prospective cohort study conducted in the city of Münster, Germany. Its main objective is to investigate the mutual relationship between depression and subclinical arteriosclerosis [24]. It is based on the examination of three cohorts: (1) Patients with depression, recruited in local psychiatric hospitals and practices, (2) patients with CVD, recruited in local cardiology departments and rehabilitation facilities, and (3) population-based controls, randomly drawn from the register of the city of Münster. Initially, 2258 subjects in the age range of 35 to 65 years were recruited for the BiDirect baseline examination (July 2010 to June 2013). The participants of all cohorts were examined in parallel with identical methods, including a computer-guided personal interview on socio-demographic characteristics, lifestyle habits, and medical history, an extensive psychiatric assessment, a clinical diagnostic work-up on vascular status, anthropometric measurements, blood samples, sensory and neuropsychological tests, as well as a magnetic resonance imaging of the brain. Further details on rational and design of the BiDirect Study have been described elsewhere [24,25]. The BiDirect Study was approved by the ethics committee of the University of Münster and the Westphalian Chamber of Physicians in Münster. All participants provided written informed consent.

For the present analysis, only participants of the depression cohort and the control cohort were included, resulting in a sample size of 1911 participants (999 patients with depression, 912 controls). Patients with depression were only included if they had a clinical diagnosis of a depressive episode (F32) or a recurrent depression (F33) according to ICD-10. Patients with a diagnosis of other affective disorders (e.g., bipolar disorder) were excluded for this analysis. Next, participants with missing data on diet, depression subtypes, depression severity, and important covariates were excluded. Overall, the final sample included 1660 subjects (840 patients with depression, 820 controls). For sensitivity analyses, we additionally excluded subjects of the control group who reported present depressive symptoms according to the Center for Epidemiologic Studies Depression Scale (CES-D, cutoff ≥16), resulting in a final subsample of 1511 subjects (840 patients with depression, 671 controls).

### Psychiatric assessment

The psychiatric assessment of the patients with depression was conducted by trained psychologists during recruitment and combined multiple scales in order to provide the classification of depression subtypes and severity. The participants answered a structured clinical interview, which included the modules A (on MDD) and A’ (on MDD with melancholic features) of the Mini International Neuropsychiatric Interview (MINI) [26], six selected questions of the Inventory of Depressive Symptomatology (IDS) on atypical features [27], as well as the Hamilton Depression Rating Scale (HAM-D, 17-item version) to assess depression severity [28]. Further characteristics on the course of depression (e.g., number of depressive episodes or hospitalizations) were noted at the start of the interview. Information on current antidepressant medication of the psychiatric patients was collected from the patients’ medical records. The basic psychiatric assessment of the controls was conducted by trained study nurses as a part of the computer-guided personal interview. If these participants showed indications for a present MDD according to the MINI, their psychiatric assessment was continued by the study psychologists. Additionally, participants of both cohorts answered the self-report Center for Epidemiologic Studies Depression Scale (CES-D, 20-item version) on depressive symptomatology [29].

Patients of the depression cohort were subsequently classified as ‘melancholic’ or ‘atypical’ subtype according to DSM-IV criteria. Participants who showed both melancholic and atypical features were categorized as ‘mixed’; participants with neither melancholic nor atypical features were categorized as ‘undifferentiated’.

### Dietary assessment

All participants answered a food frequency questionnaire (FFQ), which had been validated against 7-day weighed dietary records in the past [30]. The FFQ was part of the computer-guided personal interview. It assessed the usual intake of 18 foods by requesting intake frequencies over the last year (7 categories: several times a day, daily or almost daily, several times a week, about once a week, several times a month, once a month or less, never). The FFQ did not include information on beverage consumption, portion sizes, or detailed nutrient intakes.

In a next step, a diet quality score was calculated for each participant, using a validated diet score matrix [31]. This diet quality score reflects the adherence to the nutritional recommendations of the German Nutrition Society on a healthy and well-balanced diet, e.g., including a daily consumption of fruits, vegetables, and (whole) grain products, fish intake about once a week, a moderate intake of animal products such as meat and eggs, and a low consumption of snacks like chocolate, cake, or salty snacks [31]. The score was based on the following 15 food groups: Meat, meat products, fish, potatoes, pasta, rice, salad/raw vegetables, cooked vegetables, fruits, chocolate, cake/pastries/biscuits, salty snacks, (whole grain) bread, muesli/oat flakes/cornflakes, and eggs. Each item was scored between 0 to 2 points according to the recommended intake frequency (0 = adverse, 1 = medium, 2 = optimal intake). Thus, the overall diet quality score ranged from a minimum of 0 to a maximum of 30 points, with higher scores reflecting better diet quality.

### Assessment of covariates

During the interview, socio-demographic data on age, sex, marital status, education, and job status were recorded and categorized as follows: Marital status (married, divorced, single, widowed); education (low education, secondary education certificate, university entrance qualification, university degree); employment status (fully employed, part-time employed, unemployed, retired, other).

The interview also contained questions on the participants’ medical history. A comorbidity-index (including hypertension, myocardial infarction, stroke, diabetes mellitus, and cancer) was calculated as a linear score by summing up the number of above-mentioned diseases for each participant. Furthermore, data on current medication was collected. All reported medications were coded according to the Anatomical Therapeutic Chemical Classification System (ATC). The intake of drugs labeled with the ATC code N-06-A was used to identify participants taking antidepressant medications.

Body weight and height were measured by trained study nurses using a calibrated measuring station (Seca GmbH & Co. KG, Hamburg, Germany). The participants wore clothes but no shoes during measurement. Body mass index (BMI) was calculated as weight/height^2^ (kg/m^2^). Physical activity was assessed by the short version of the International Physical Activity Questionnaire (IPAQ) [32]. Physical activity levels as well as continuous values of MET-minutes per week (MET = metabolic equivalent of task) were calculated according to the IPAQ scoring protocol. Furthermore, participants reported on their current smoking status (current smoker, former smoker, or nonsmoker).

### Statistical analyses

Descriptive data are presented as number of participants (percentage) for categorical variables or as median (1^st^ quartile; 3^rd^ quartile) for continuous variables. Differences in baseline characteristics across groups were tested using Chi-Square-Test for categorical variables and Mann-Whitney-U-Test for continuous ones.

Adjusted means of diet quality scores were calculated and group differences were tested using analysis of covariance (ANCOVA). Potential confounders that were added into the final models were identified either through literature, descriptive analyses, or a significant association with the outcome of interest. The analyses examining differences in diet quality scores between controls and patients with depression/subtypes of depression were adjusted for sex and age in the basic model (model 1) and additionally adjusted for marital status, education, job status, BMI, physical activity, smoking status, comorbidities, and antidepressant medication in the final model (model 2). Tests of interaction and additional sex-stratified analyses showed no differences between genders. Thus, we conducted the following analyses without sex-stratification. The analysis of the association between depression severity and diet quality was conducted stratified for controls and patients with depression, using linear regression models that were adjusted for the same covariates as mentioned above (except antidepressant medication).

All statistical analyses were performed in SAS (SAS 9.4; SAS Institute, Cary, North Carolina, USA). All p-values were two-tailed and values of <0.05 were considered as significant.

## Results

The descriptive characteristics of patients with depression and controls are presented in Table 1. The group of controls included fewer women, was slightly older, showed a higher level of education, and was more often employed compared to the subjects with depression. With regard to lifestyle habits, patients with depression consistently reported more unhealthy behaviors: They had higher BMI values, reported less physical activity, and the proportion of current smokers was more than twice as high compared to the control group. Patients with depression also reported more comorbidities.

**Table 1:** **Characteristics of the study participants** (**BiDirect Study**, **n** = **1660**)

**Characteristics**	**BiDirect cohorts**		**p** ^**a**^
	**Population**-**based controls**	**Patients with depression**	
	(**n** = **820**)	(**n** = **840**)	
**Socio**-**demographic characteristics**			
Women, n (%)	414 (50.5)	497 (59.2)	<0.001
Age, years	53.5 (46.3; 59.4)	49.4 (43.9; 55.6)	<0.001
Married, n (%)	531 (64.8)	514 (61.2)	0.13
Employed, n (%)	655 (79.9)	559 (66.6)	<0.001
University degree, n (%)	326 (39.8)	196 (23.3)	<0.001
**Lifestyle factors**			
BMI, kg/m^2^	26.1 (23.5; 29.4)	27.9 (24.5; 31.6)	<0.001
Physical activity, MET-min. per week	2302 (1181; 4259)	1746 (878; 3360)	<0.001
Current smoker, n (%)	175 (21.3)	363 (43.2)	<0.001
**Comorbidities**, n (%)			
History of diabetes mellitus	27 (3.3)	57 (6.8)	0.001
History of hypertension	224 (27.3)	304 (36.2)	<0.001
History of myocardial infarction	16 (2.0)	14 (1.7)	0.66
History of stroke	11 (1.3)	18 (2.1)	0.21
History of cancer	52 (6.3)	54 (6.3)	0.94
History of at least one of these comorbidities	272 (33.2)	364 (43.3)	<0.001
**Psychiatric characteristics**			
Depression severity, CES-D score	8 (4; 14)	29 (18; 37)	<0.001
Depression severity, HAM-D score		14 (10; 19)	
Use of antidepressants, n (%)		514 (61.2)	
Subtypes of depression			
Melancholic		516 (61.4)	
Atypical		42 (5.0)	
Mixed		83 (9.9)	
Undifferentiated		199 (23.7)	

Data are presented as number of participants (percentage) for categorical variables or as median (1^st^ quartile; 3^rd^ quartile) for continuous variables. Total percentages may not add up to 100 % because of rounding.

Abbreviations: BMI, body mass index; CES-D, Center for Epidemiologic Studies Depression Scale; HAM-D, Hamilton Depression Rating Scale; MET, metabolic equivalent of task.

^a^Differences between cohorts were tested using Chi-Square-Test for categorical variables and Mann-Whitney-U-Test for continuous variables.

The participants’ diet quality scores ranged from a minimum of 4 to a maximum of 27 points. The comparison of adjusted diet quality scores between controls and patients with depression is presented in Table 2. In the basic model (adjusted for age and sex), controls and patients with depression had very similar diet quality scores (controls: 14.7; depression: 14.6; p = 0.44). After adjustment for further covariates, the diet quality score of the control group was slightly attenuated. However, the scores of both groups still remained similar (controls: 14.2; depression: 14.5; p = 0.22). The exclusion of controls with depressive symptoms (CES-D ≥16) did not change the actual results (controls: 14.4; depression: 14.5; p = 0.64 in the final model). We conducted additional sensitivity analyses and stratified for BMI categories and physical activity levels, since the relationship between mood, nutrition, physical activity, and weight is a very complex one [33,34]. However, we found no interactions for any of these factors (data not shown). On closer examination of intake frequencies in terms of specific food groups, we found some significant differences between controls and patients with depression: For instance, controls reported higher intakes of fruits, chocolate, cake, and pasta, as well as lower intakes of meat and poultry compared to the group of patients with depression (Additional file 1: Figure S1).

**Table 2:** **Adjusted means** ^**a**^ **of diet quality scores stratified by cohorts** (**BiDirect Study**)

	**BiDirect cohorts**		**p**
	**Population**-**based controls**	**Patients with depression**	
**Total sample** **(n** = **1660)**	n = 820	n = 840	
**Model 1** ^**b**^	14.7 (14.5 - 15.0)	14.6 (14.3 - 14.8)	0.44
**Model 2** ^**c**^	14.2 (13.8 - 14.7)	14.5 (14.2 - 14.9)	0.22
**Sensitivity analysis** ^**d**^ **(n** = **1511)**	n = 671	n = 840	
**Model 1** ^**b**^	14.9 (14.6 - 15.1)	14.6 (14.3 - 14.8)	0.13
**Model 2** ^**c**^	14.4 (13.9 - 14.8)	14.5 (14.1 - 14.9)	0.64

^a^Adjusted means (95% confidence interval) were obtained and group differences were tested using analysis of covariance (ANCOVA).

^b^Model 1 adjusted for age and sex.

^c^Model 2 further adjusted for marital status, education, job status, smoking status, body mass index, physical activity, comorbidities, and antidepressant intake.

^d^After exclusion of controls with present depressive symptoms indicated by CES-D ≥16 (n = 149).

Table 3 presents the adjusted mean diet quality scores for controls and patients with different subtypes of depression. From a total of 840 patients with depression, 516 (61.4 %) were classified as ‘melancholic’, 42 (5.0 %) as ‘atypical’, 83 (9.9 %) as ‘mixed’, and 199 (23.7 %) as ‘undifferentiated’. In the basic as well as in the final model, we found significant differences in diet quality scores between the groups (p = 0.001). In both models, patients with melancholic depression showed the highest, whereas patients with atypical depression showed the lowest diet quality scores. A pairwise comparison of the atypical and the melancholic subtype revealed significant differences in overall diet quality (melancholic: 14.9; atypical: 13.7; p = 0.03 in the final model). A more detailed analysis regarding specific food groups supports this finding. We found significant differences in intake frequencies of the food groups ‘chocolate’ (p = 0.02) and ‘cake/pastries/biscuits’ (p = 0.02) between the melancholic and the atypical depression subtype (Additional file 1: Figure S2). Patients with atypical depression reported higher intake frequencies of chocolate (daily or several times per week: 66.7 %) and cake/pastries/biscuits (daily or several times per week: 54.8 %) than patients with melancholic depression (48.5 % and 35.1 %, respectively). Compared to the control subjects, patients with undifferentiated, atypical, and mixed depression had lower diet quality scores, but these differences were not statistically significant in the final model. In contrast, patients with melancholic depression had significantly higher diet quality scores than controls (controls: 14.2; melancholic: 14.9; p = 0.005 in the final model). Exclusion of controls with depressive symptoms (CES-D ≥16) yielded very similar results.

**Table 3:** **Adjusted means** ^**a**^ **of diet quality scores stratified by depression subtypes** (**BiDirect Study**)

	**Population-** **based controls**	**Patients with depression by subtypes**								**Overall p**	**Contrast p** ^**f**^ **(atypical vs. melancholic)**
		**Melancholic**	**p** ^**e**^	**Atypical**	**p** ^**e**^	**Mixed**	**p** ^**e**^	**Undifferentiated**	**p** ^**e**^		
**Total sample** (**n** = **1660**)											
	n = 820	n = 516		n = 42		n = 83		n = 199			
**Model 1** ^**b**^	14.7 (14.5 - 15.0)	15.0 (14.7 - 15.3)	0.19	13.8 (12.8 - 14.9)	0.13	14.0 (13.2 - 14.8)	0.08	13.9 (13.4 - 14.4)	0.004	0.001	0.05
**Model 2** ^**c**^	14.2 (13.8 - 14.6)	14.9 (14.5 - 15.3)	0.005	13.7 (12.6 - 14.8)	0.38	14.0 (13.2 - 14.8)	0.56	14.0 (13.4 - 14.5)	0.43	0.001	0.03
**Sensitivity analysis** ^**d**^ **(n** = **1551)**											
	n = 671	n = 516		n = 42		n = 83		n = 199			
**Model 1** ^**b**^	14.9 (14.6 - 15.1)	15.0 (14.7 - 15.3)	0.60	13.8 (12.7 - 14.9)	0.07	14.0 (13.2 - 14.7)	0.04	13.9 (13.4 - 14.4)	0.001	<0.001	0.05
**Model 2** ^**c**^	14.3 (13.8 - 14.8)	14.9 (14.4 - 15.3)	0.05	13.7 (12.6 - 14.8)	0.27	13.9 (13.1 - 14.7)	0.33	13.9 (13.4 - 14.5)	0.21	0.003	0.04

^a^Adjusted means (95% confidence interval) were obtained and group differences were tested using analysis of covariance (ANCOVA).

^b^Model 1 adjusted for age and sex.

^c^Model 2 further adjusted for marital status, education, job status, smoking status, body mass index, physical activity, comorbidities, and antidepressant intake.

^d^After exclusion of controls with present depressive symptoms indicated by CES-D ≥16 (n = 149).

^e^Contrast p-values: Pairwise comparisons between subtypes and controls (=reference).

^f^Contrast p-value: Pairwise comparison between atypical and melancholic (=reference) depression subtype.

Results on the association between depression severity and diet quality are presented in Table 4. The median CES-D score was 8 points for controls and 29 points for patients with depression; additionally, the patients with depression showed a median HAM-D score of 14 points (see Table 1). Among patients with depression, no association between depression severity and diet quality was observed, neither for severity assessment by CES-D (self-reported) nor by HAM-D (interview-based). Among controls, the inverse association between higher depressive symptoms and lower diet quality in the basic model (β = −0.032, p = 0.03) was explained by additional covariates in the final model (β = −0.019, p = 0.21).

**Table 4:** **Associations between depression severity and diet quality stratified by controls and patients with depression** (**BiDirect Study**)

	**Regression-** **coefficient** **(standard error)**	**p**
**Controls** **(n** = **820)**		
**CES**-**D score** **(self**-**reported)**		
Model 1^a^	−0.032 (0.015)	0.03
Model 2^b^	−0.019 (0.015)	0.21
**Patients with depression** **(n** = **840)**		
**CES**-**D score** **(self**-**reported)**		
Model 1^a^	−0.005 (0.011)	0.68
Model 2^b^	0.007 (0.011)	0.52
**HAM**-**D score** (**interview**-**based**)		
Model 1^a^	−0.003 (0.020)	0.90
Model 2^b^	0.016 (0.021)	0.45

Abbreviations: CES-D, Center for Epidemiologic Studies Depression Scale; HAM-D, Hamilton Depression Rating Scale.

^a^Model 1 adjusted for age and sex.

^b^Model 2 further adjusted for marital status, education, job status, smoking status, body mass index, physical activity, and comorbidities.

## Discussion

In the present study, we examined differences in diet quality between a large cohort of patients with a clinical diagnosis of depression and healthy population-based controls. We found no significant differences between both groups if depression was considered as one disease entity. However, we observed significant differences between patients with different depression subtypes. There was no association between depression severity and diet quality.

Prior research on the effect of depression on overall diet provided inconsistent results [10]. For the most part, previous studies utilized depression scales to assess depressive symptomatology. Some of these studies suggested that the presence of depressive symptoms was associated with lower overall diet quality [35-38], others did not support this association [39]. Only few studies examined diet in samples with a clinical diagnosis of depression [14,40]. For example, Kilian et al. investigated health behaviors of psychiatric patients in comparison with a sample of the German general population. They found that patients with depression showed a significantly increased number of unhealthy food habits [14]. In contrast, Beydoun and Wang did not find an association between depression and diet quality [40]. All of the above-mentioned studies were cross-sectional in design and none of them considered any subtypes of depression. However, there is also compelling evidence for the other direction of this relationship, namely dietary patterns predicting the development of depression. Several high-quality longitudinal studies showed that healthy and Mediterranean dietary patterns may have a protective effect against depression, whereas unhealthy Western dietary patterns may increase the depression risk [41,42]. Recent systematic reviews and meta-analyses came to similar conclusions [10-13].

In our study, we did not find differences in diet quality between controls and patients with depression in general, but we did find differences after considering distinct depression subtypes. The direct comparison between patients with melancholic and atypical depression revealed significant differences in their overall diet quality, for instance, due to higher intakes of chocolate or cake/pastries/biscuits. These novel results imply that patients with atypical depression may consume more energy-dense low-quality foods such as sweets or snacks, presumably due to their increased appetite. In contrast, patients with melancholic depression seem to consume less of these low-quality foods, which in turn has positive impact on their actual diet quality.

The underlying mechanisms that link depression and eating behavior may include sensory, physiological, as well as psychological pathways [5]. One recent study suggested emotional eating as one explaining factor [17]. Emotional eating means the tendency to preferably consume energy-dense sweet and high-fat foods in response to negative affects and stress [17]. Several studies confirmed that the presence of depressive symptoms is associated with emotional eating [17,39,43], but there is lack of evidence regarding the role of specific depression symptom profiles on emotional eating. In a study of Parker and Crawford, they observed that craving for comfort foods, such as chocolate or cake, increased with increasing number of atypical depressive symptoms and identified rejection sensitivity as one important predictor of such craving [44]. It seems conceivable that symptoms such as mood reactivity and rejection sensitivity, which are both related to atypical depression according to DSM-IV criteria, may be associated with emotional eating patterns in a special way and, thus, may predict unhealthy food choices and poorer diet quality. However, the underlying biological pathways are not sufficiently understood, especially with respect to distinct subtypes of depression. Different biological correlates (e.g., hormones, neurotransmitters, or inflammatory cytokines) have been proposed to influence appetite regulation and eating behavior [45-47]. However, study results on biological correlates in distinct subtypes of depression are inconsistent and partly conflicting up to now [47]. Hence, further research is needed to understand the underlying biological mechanisms that alter appetite and food intake in different subtypes of depression.

In our study, we did not observe an association between depression severity and diet quality. Only few studies examined the effect of depression severity on diet [34,35,39]. Overall, these studies also reported inconsistent results. Appelhans et al. observed that higher depression severity (assessed by Beck Depression Inventory II) was associated with lower diet quality in a sample of obese subjects with major depression [34]. Beydoun et al. also reported an inverse association between higher depressive symptoms (assessed by CES-D) and lower diet quality in a representative sample of US adults [35]. In line with our results, Whitaker et al. found no association between depressive symptoms (assessed by CES-D) and overall diet [39].

Our study has several strengths. First, it included subjects with a clinical diagnosis of depression assessed by clinical interview, which is known to be the gold standard in depression assessment [19,20]. Next, the BiDirect Study also includes a sample of randomly drawn controls from the same sampling area and in the same age range as the patients. Furthermore, our study extends previous research by investigating different depression subtypes. To our knowledge, the present study was the first one to consider distinct subtypes in regard to the relationship between depression and overall diet. Finally, due to the broad examination program of the BiDirect Study, we were able to adjust the analyses for a wide range of important covariates, including socio-demographic variables, lifestyle factors, and health-related characteristics.

Despite these strengths, the study has some limitations that should be noted when interpreting the results. The present analysis was based on cross-sectional baseline data. Hence, the actual direction of the relationship between diet and depression cannot be determined. Longitudinal studies that provide superior evidence to cross-sectional studies have also found associations of dietary patterns predicting the development of depression [41,42]. Furthermore, observational studies like the BiDirect Study always imply the possibility of (residual) confounding. The selection of potential confounders is often challenging and there is also the risk of overadjustment, in particular, if the role of specific variables concerning the relationship of interest is not completely clear. Next, the sample sizes of some subtype groups (e.g., atypical depression) were relatively small and made it difficult to detect significant effects. Another limitation might arise from the dietary assessment method. We used a short FFQ, which contained information on the 18 most important food groups, but lacked information on portion sizes, energy intake, and beverage consumption (for instance, on sweetened soft drinks that may be a contributor to poor health conditions themselves [48]). Compared to more comprehensive FFQ used in specific nutritional epidemiologic studies, this is a rather short tool and allows only crude estimations of overall diet. Since we had no information on detailed nutrient intakes, we could not use most of the other diet scores that are known from literature (e.g., Healthy Eating Index, Diet Quality Index), because these often need both food group-based as well as nutrient-based information. However, validity studies reported that our FFQ and diet quality score had adequate validity on group level [30,31]. The present diet quality score shows many similarities to other scores or indices, e.g., as it also assumes fruits, vegetables, (whole) grain products, and fish to be high-quality foods, considers animal products as foods that should be consumed in moderation, and rates sweets and salty snacks to be of rather low quality. Overall, these assumptions are similar to the general idea of most diet scores. Also, dietary assessment methods that rely on participants’ self-reported information are always prone to misreporting. Moreover, it may be possible that depression itself is associated with reporting accuracy, e.g., by influencing cognitive functioning or the response behavior in terms of social desirability [49]. Finally, a difference in diet quality scores of 1 to 2 points may be small and its clinical or daily relevance remains unclear.

## Conclusions

In conclusion, the present study did not find significant differences in diet quality between population-based controls and patients with depression if depression was considered as one entity, but did find differences after considering different subtypes of depression. Compared to a concept that views depression as a homogeneous disease entity, the consideration of distinct subtypes seems to be a promising approach to analyze the role of specific symptom profiles on diet. However, diet quality as assessed by simple scores or indices may be a limited approach to investigate the relationship between diet and mental health. Additional prospective studies with more precise dietary assessment methods are needed to further clarify this relationship.

## Additional file

Additional file 1: Figure S1. Mean intake frequencies of food groups, stratified by cohorts (BiDirect Study). **Figure S2.** Mean intake frequencies of food groups, stratified by controls and depression subtypes (BiDirect Study).
