# Supplementary material for: Associations between depression subtypes, depression severity and diet quality: cross-sectional findings from the BiDirect Study
Source: BMC Psychiatry. 2015 Mar 4;15:38. doi: 10.1186/s12888-015-0426-9 (PMC4355144; doi:10.1186/s12888-015-0426-9)
Supplement: Additional file 1: Figure S1. — Mean intake frequencies of food groups, stratified by cohorts (BiDirect Study). Figure S2. Mean intake frequencies of food groups, stratified by controls and depression subtypes (BiDirect Study). [file 12888_2015_426_MOESM1_ESM.pdf]

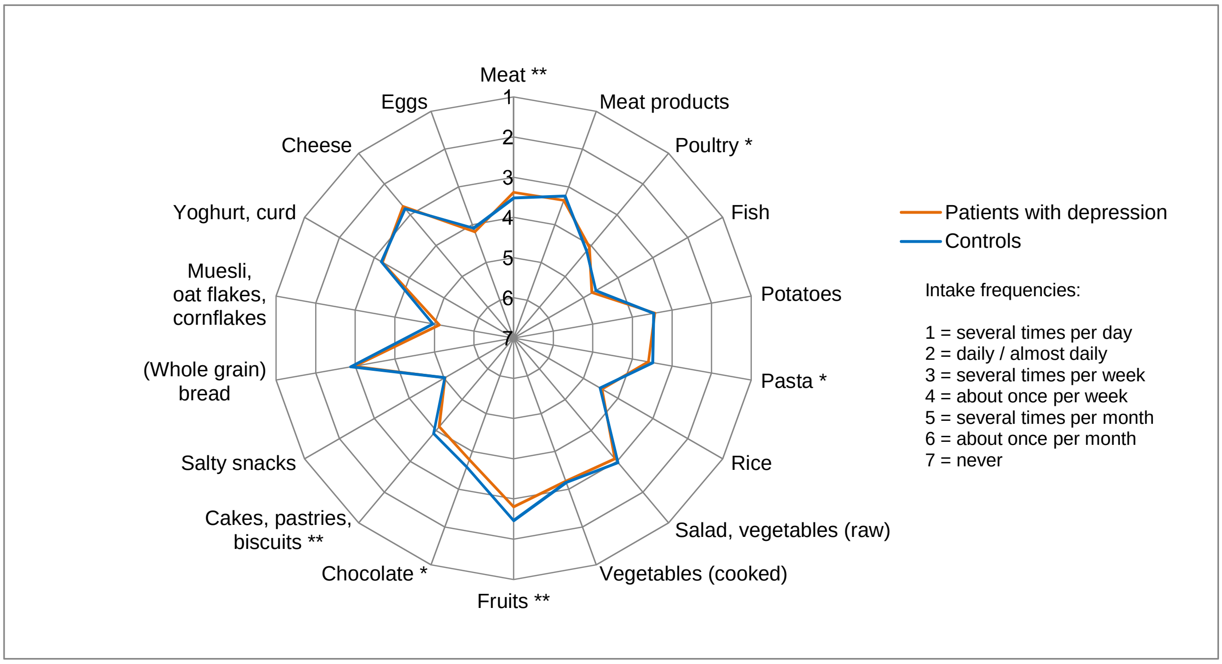

Figure 1 Mean intake frequencies of food groups, stratified by cohorts (BiDirect Study)

Differences in intake frequencies between controls (n=820) and patients with depression (n=840) were tested using Mann-Whitney-U-Test (* p<0.05; ** p<0.01).

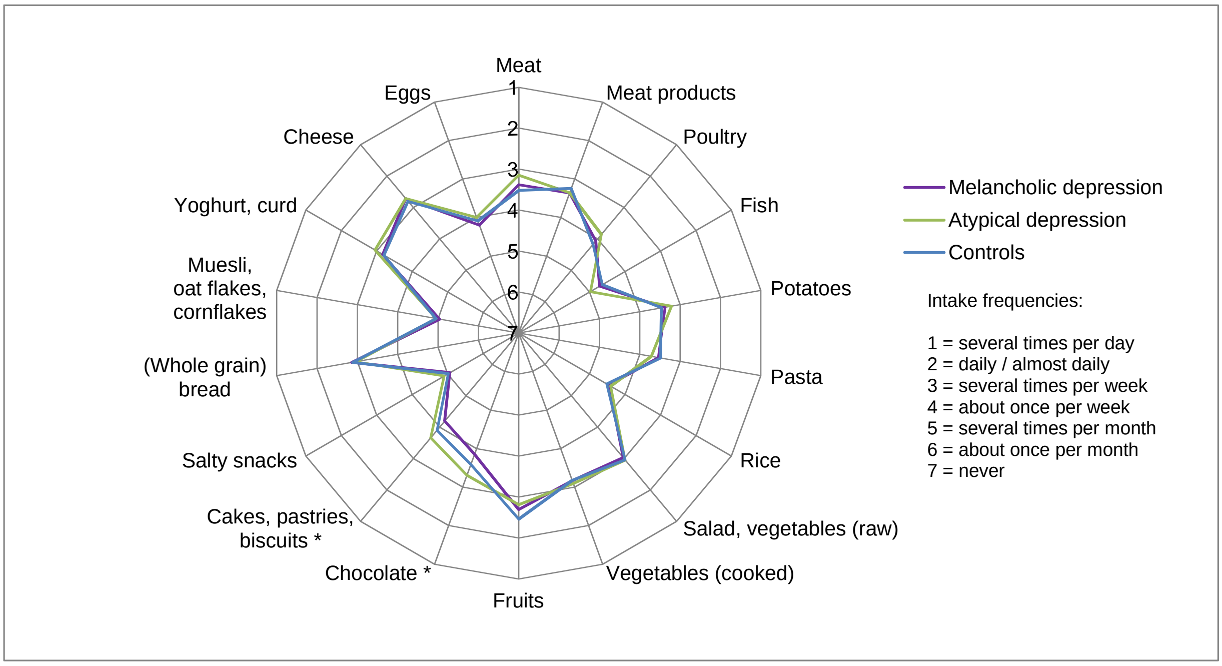

Figure 2 Mean intake frequencies of food groups, stratified by controls and depression subtypes (BiDirect Study)

Differences in intake frequencies between participants with melancholic depression (n=516) and with atypical depression (n=42) were tested using Mann-Whitney-U-Test (* p<0.05; ** p<0.01).
